# Integration of high-throughput omics technologies in medicinal plant research: The new era of natural drug discovery

**DOI:** 10.3389/fpls.2023.1073848

**Published:** 2023-01-18

**Authors:** Wenting Zhang, Yuan Zeng, Meng Jiao, Chanjuan Ye, Yanrong Li, Chuanguang Liu, Jihua Wang

**Affiliations:** ^1^Guangdong Provincial Key Laboratory of Crops Genetics & Improvement, Crops Research Institute, Guangdong Academy of Agricultural Sciences, Guangzhou, China; ^2^Guangdong Provincial Engineering & Technology Research Center for Conservation and Utilization of the Genuine Southern Medicinal Resources, Guangzhou, China; ^3^School of Plant and Environmental Sciences, Virginia Tech, VA, Blacksburg, United States; ^4^Southern Piedmont Agricultural Research and Extension Center, Virginia Tech, VA, Blackstone, United States; ^5^College of Life Sciences, South China Agricultural University, Guangzhou, China; ^6^Rice Research Institute, Guangdong Rice Engineering Laboratory, Guangdong Key Laboratory of New Technology in Rice Breeding, Guangdong Academy of Agricultural Sciences, Guangzhou, China

**Keywords:** medicinal plant, high-throughput omics, biosynthesis pathways, active ingredients, phytochemicals

## Abstract

Medicinal plants are natural sources to unravel novel bioactive compounds to satisfy human pharmacological potentials. The world’s demand for herbal medicines is increasing year by year; however, large-scale production of medicinal plants and their derivatives is still limited. The rapid development of modern technology has stimulated multi-omics research in medicinal plants, leading to a series of breakthroughs on key genes, metabolites, enzymes involved in biosynthesis and regulation of active compounds. Here, we summarize the latest research progress on the molecular intricacy of medicinal plants, including the comparison of genomics to demonstrate variation and evolution among species, the application of transcriptomics, proteomics and metabolomics to explore dynamic changes of molecular compounds, and the utilization of potential resources for natural drug discovery. These multi-omics research provide the theoretical basis for environmental adaptation of medicinal plants and allow us to understand the chemical diversity and composition of bioactive compounds. Many medicinal herbs’ phytochemical constituents and their potential health benefits are not fully explored. Given their large diversity and global distribution as well as the impacts of growth duration and environmental factors on bioactive phytochemicals in medicinal plants, it is crucial to emphasize the research needs of using multi-omics technologies to address basic and applied problems in medicinal plants to aid in developing new and improved medicinal plant resources and discovering novel medicinal ingredients.

## Introduction

Many medicinal plant-derived alkaloid, terpene, polyphenol, coumarin, and saponin have received increasing attentions from the pharmaceutical industries due to their potent antioxidant, antibacterial, antiphlogistic, anticancer, and antidiabetic activities (Mumtaz et al., 2017; Pandita et al., 2021). However, large scale production of these active ingredients was limited due to the lack of medicinal plant genomic information. As the advancement of high-throughput sequencing technology, genomes of many medicinal plants are assembled, leading to functional characterization of genes that involve in specific secondary biosynthesis pathways. For example, genes in the biosynthesis pathway of heterologously producing saponin, a biological compound displaying antimicrobial and anti-inflammatory activities, were characterized following the publication of Chinese ginseng (*Panax notoginseng*) genomes (Fan et al., 2020; Jiang et al., 2021). The biosynthesis, regulation, and transportation of a bioactive component, benzylisoquinoline alkaloid (BIA) in landraces of Chinese opium poppy was revealed upon the availability of its genome (Hu et al., 2018).

Incorporating other high-throughput sequencing or analytical techniques in metabolomics, proteomics, and transcriptomics into medicinal plant research can aid in discovering functional genes, key metabolites, biological elements with pharmacological potential and molecular markers associated with phytochemical compounds. For example, mining transcriptome and metabolome profiles of mayapple (*Podophyllum hexandrum*), a plant with anticancer compounds, identified genes involved in biosynthetic pathway of podophyllotoxin (Lau and Sattely, 2015). *Abelmoschus esculentus* is a medicinal plant containing a large amount of active ingredients such as anthocyanins, flavonoids, polysaccharides, and terpenoids. Based on the transcriptome data of *A. esculentus*, one significant marker was detected by association analysis of fruit color (anthocyanin content) which may be used for the genetic improvement of *A. esculentus* (An et al., 2022). Analyzing proteomic datasets of *Dendrobium huoshanense* led to discovery of crotonylated proteins in the Calvin cycle, photosynthesis, and alkaloid and polysaccharide biosynthesis metabolic pathways (Wu et al., 2022a).

To date, multi-omics approaches have promoted the development of large databases on a specific medicinal plant species or multiple species spanning their genome, transcriptome, proteome, metabolome, or phytochemicals (**Table 1**). For example, Ginseng Genome Database is the first comprehensive database among other herbs due to its well-established genome information (Jayakodi et al., 2018). The Global Pharmacopoeia Genome Database (GPGD) is a database contains 2,203 organelle genomes from 674 species, 55 whole genomes from 49 species, and 9,682 transcriptome datasets from 350 species (Liao et al., 2021). MepmiRDB, the first MicroRNA (miRNA) database of medicinal plants, gathers miRNA information of 29 species such as ginseng, wolfberry and red sage (Yu et al., 2019). Moreover, phytochemical databases of medicinal plants, such as IMPPAT (Mohanraj et al., 2018), NPACT (Mangal et al., 2013), NuBBEDB (Pilon et al., 2017), and NANPDB (Ntie-Kang et al., 2017), are also publicly available. Each phytochemical database provides classic information (*e.g.*, common name, taxonomy, location, medicinal parts, and application) and chemical/structure information of medicinal properties. Together, these databases allow researchers to conduct deep data mining to explore gene annotation and expression profiles of pharmacological properties, study the roles of miRNA in regulating biosynthesis and accumulating secondary metabolites, and acquire DNA barcode data to facilitate identification of medicinal materials.

**Table 1:** Databases on medicinal plant -omics and phytochemicals.

Database name	Database type	URL	Genus/Species name	Reference
Specific genus/species				
LjaFGD	Genome, Transcriptome	http://www.gzybioinformatics.cn/LjaFGD/index.php	*Lonicera japonica*	(Xiao et al., 2021)
RPGD	Genome, Transcriptome	http://bioinfor.kib.ac.cn/RPGD/	*Rhododendron*	(Liu et al., 2021b)
AprGPD	Genome, Transcriptome	http://apricotgpd.com	*Prunus*	(Chen et al., 2021)
croFGD	Genome, Transcriptome	http://bioinformatics.cau.edu.cn/croFGD/	*Catharanthus roseus*	(She et al., 2019)
MGH	Genome, Transcriptome	http://maca.eplant.org	*Lepidium meyenii*	(Chen et al., 2018)
SmGDB	Genome, Transcriptome	http://8.140.162.85/	*Salvia miltiorrhiza*	(Zhou et al., 2022)
Ginseng Genome Database	Genome, Transcriptome, Proteome, Metabolome	http://ginsengdb.snu.ac.kr/	*Panax ginseng*	(Jayakodi et al., 2018)
Comprehensive species				
HMOD	Genome, Transcriptome, Metabolome	http://herbalplant.ynau.edu.cn/	Up to 138 species	(Wang et al., 2018)
BPGD	Genome, Transcriptome	http://www.bpgenome.com	Up to 34 species	(Zhou et al., 2021a)
GPGD	Genome, Transcriptome	http://www.gpgenome.com	Up to 350 species	(Liao et al., 2021)
MPOD	Genome, Transcriptome	http://medicinalplants.ynau.edu.cn/	Up to 187 species	(He et al., 2022b)
TCMPG	Genome	http://cbcb.cdutcm.edu.cn/TCMPG/	195 species	(Meng et al., 2022)
1 K-MPGD	Genome	http://www.herbgenome.com/	108 species	(Su et al., 2022)
MepmiRDB	miRNA	http://mepmirdb.cn/mepmirdb/index.html	29 species	(Yu et al., 2019)
IMPPAT	Phytochemicals	https://cb.imsc.res.in/imppat	–	(Mohanraj et al., 2018)
NPACT	Phytochemicals	http://crdd.osdd.net/raghava/npact/	–	(Mangal et al., 2013)
AromaDb	Phytochemicals	http://bioinfo.cimap.res.in/aromadb/	–	(Kumar et al., 2018)
NANPDB	Phytochemicals	http://african-compounds.org/anpdb/	–	(Ntie-Kang et al., 2017)
NuBBEDB	Phytochemicals	https://nubbe.iq.unesp.br/portal/nubbedb.html	–	(Pilon et al., 2017)
ETM-DB	Phytochemicals	http://biosoft.kaist.ac.kr/etm/home.php/	–	(Bultum et al., 2019)

-: not applicable.

Although the rapid advancement of sequencing and spectrometry technologies and the availability of computational tools has stimulated medicinal plant research worldwide, there is still not a comprehensive review from a multi-omics aspect summarizing the current research progress in discovering genes, proteins, and key/secondary metabolites that involve in biosynthesis pathways in medicinal plants. Therefore, our goal for this review is to provide an update on application of omics technologies in medicinal plant research to explore compounds in biosynthesis pathways for natural drug discovery. We briefly discuss medicinal plant genomes at chromosome, chloroplast, and mitochondrial level, and the needs of conducting comparative genomics, epigenomics and pan-genomics research as well as constructing genetic mapping for the development and selection of medicinal plants with high bioactive compounds. Next, we emphasize the importance of applying other omics approaches (*i.e.*, transcriptome, proteome and metabolome) to identify key molecular products related to biosynthesis of active ingredients of medicinal plants, with a focus on phenolic acid, flavonoids, and alkaloid biosynthesis pathways. Under the current challenges in medicinal plant breeding, we hope that our review could inspire more efforts to integrate high-throughput omics technologies in medicinal plant research to facilitate natural drug discovery.

## Updates in medicinal plant genomes

Medicinal plant genome assembly is challenging due to their large genome sizes and complicated polyploid chromosomes; however, long-read sequencing technologies have increased a significant number of assembled medicinal plant genomes at the chromosomal level (**Supplementary Table 1**; as the date of July 30^th^, 2022). According to Cheng et al. (2021), 161 reference genomes representing 126 medicinal plant species (in red in **Supplementary Table 1**) were published as the date of June 4, 2021. In this review, we summarized additional 118 genomes from 78 medicinal plant species, and they are presented in **Supplementary Table 1** (in black).

**Figure 1:**
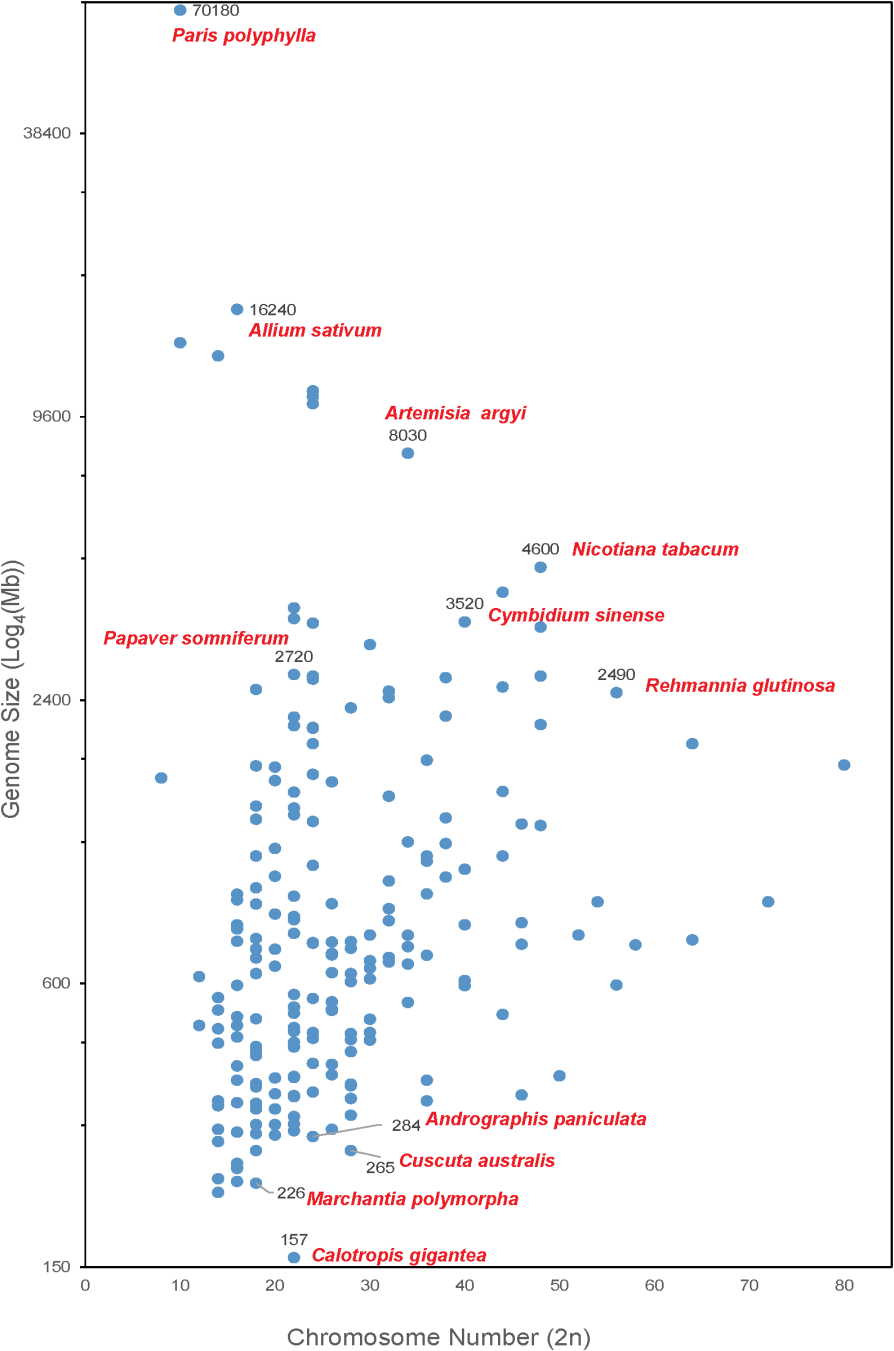
Medicinal plants with complete genome sequencing. The X-axis shows the chromosome number of a medicinal plant genome. The Y-axis represents the genome size (Log_4_(Mb)) of each plant.

As shown in **Figure 1**, the chromosome number of medicinal plants varies widely, ranging from 8 to 80, and the genome sizes are from 157 Mb (crown flower, *Calotropis gigantea*) (Hoopes et al., 2018) to 70.18 Gb. The largest genome is *Paris polyphylla* (Li et al., 2020b) followed by the well-known vegetable *Allium sativum* (Garlic) (Liu et al., 2021a) with a genome size of 16.24 Gb. There are 40 plants with genome sizes between 2 to 16 Gb, including opium poppy (*Papaver somniferum-*2,720 Mb) (Pei et al., 2021b), Chinese mugwort (*Artemisia argyi-*8,030 Mb) (Miao et al., 2022), *Cymbidium sinense* (3,520 Mb) (Yang et al., 2021), cultivated tobacco (*Nicotiana tabacum-*4,600 Mb) (Sierro et al., 2014) and rehmannia (*Rehmannia glutinosa-*2,490 Mb) (Ma et al., 2021). Most of medicinal plants (*i.e.*, 232 out of 279 reported medicinal plant genomes) have a genome size smaller than 2 Gb. Of these, liverworts (*Marchantia polymorpha-*226 Mb), green chiretta (*Andrographis paniculate-*284 Mb), and Australian dodder (*Cuscuta australis-*265 Mb) are common medicinal plants with small genomes.

Plant organelles such as plastid and mitochondrion retain their own genome architectures (Wu et al., 2020). These organelles also participate in biosynthesizing fatty acids, amino acids, hormones, vitamins, and nucleotides (Dobrogojski et al., 2020), and their slow evolution due to low recombination rates are ideal for studying phylogenetic relationships and important traits (*e.g.*, phytochemicals) among medicinal plant species (Drouin et al., 2008; Smith, 2015). Currently, there are more than 2,000 chloroplast and mitochondrial genomes of medicinal plants, with only a few complete mitochondrial genomes (Wu et al., 2020; Liao et al., 2021). Following the availability of mitochondrial and chloroplast genome sequences, many medicinal plant molecular markers have been developed to aid in breeding and examining the authenticity and quality of herbs. However, there is a significant gap of using these genomic resources to illustrate functional genes in medicinal plant phytochemical production.

## Advancing comparative genomics, genetic mapping, pan-genome, and epigenome research to aid in production of secondary metabolites for pharmaceutical use

The number of comparative genomic studies to identify biosynthesis-related genes of medicinal plants has increased in the past few years. For example, genes that encode oligopeptide transporters (OPT) were shown to mediate the transportation of many bioactive chemical compounds in *Panax ginseng* and other flowering plants through conducting genomic comparisons (Su et al., 2019). Comparing genomes of *Senna tora* and other 14 plants in Fabaceae revealed that the *Senna tora* genome is enriched in biosynthesis-related genes of phenylpropane, isoflavones, and terpenes (Kang et al., 2020). Comparative genomic analysis of multiple *Cannabis sativa*, including the female strains PK with high delta-9-tetrahydrocannabinol (Δ9-THC, or simply THC), the CBDRx (cs10) strains with high cannabidiol (CBD) and female strains Cannbio-2 with balanced CBD : THC cannabinoid ratios suggested extensive copy number variation in cannabinoid synthesis (McKernan et al., 2020). These comparative genomic studies have set up good examples for researchers to explore subtle genetic variations on the plant-derived metabolite contents in other medicinal plant species, such as Chinese pistache (*Pistacia chinensis*), Ginger (*Zingiber officinale*), green chiretta (*Andrographis paniculata*) and passion fruit (*Passiflora edulia*) (**Supplementary Table 1**).

Constructing genetic linkage map provides the basis for gene mapping and cloning as well as studying the structure and function of a genome. However, the long growth cycle, short cultivation history, complex genetic background, and highly heterozygous genes of most medicinal plants make it difficult to establish their genetic maps. Traditional molecular markers like SSR, AFLP, RFLP, RAPD, and EST-SSR were used to construct genetic maps of medicinal plants such as *Trifolium pratense* (Isobe et al., 2003), artichoke (*Cynara scolymus*) (Lanteri et al., 2006), bladder campion (*Silene Vulgaris*) (Bratteler et al., 2006) and passion fruit (*Passiflora Edulis*) (Carneiro et al., 2002). Recently, genome-wide molecular markers are rapidly developed, leading to the development of a high-saturate and versatile molecular linkage maps of medicinal plants. With the increasing quantity and quality of medicinal plant genomes, we foresee that constructing genetic maps of phenotypes related to bioactive compounds in medicinal plants will bloom.

Current pan-genome and epigenome research of medicinal plants mainly focuses on determining phenotypic and environmental adaptability among subspecies. As an example, agronomic trait-associated SNPs identified by pan-genome analysis indicated that location and year significantly affected yield-related phenotypes in pigeon pea (*Cajanus cajan*), a plant species with rich vitamin B, protein, ascorbic acid and carotene (Zhao et al., 2020). Developmental methylome of the medicinal plant Cape periwinkle (*Catharanthus roseus*) determined that cellular and physiological functions are likely to be impacted by tissue-specific covariations between context-dependent DNA methylation (Dugé de Bernonville et al., 2020). The cold environment affected ginsenosides accumulation in perennial American ginseng (*Panax quinquefolium*), and a cyclically reversible dynamism between methylation and demethylation of DNA was also reported in response to temperature seasonality (Hao et al., 2020). Further investigations of medicinal plant pan-genomes and epigenomes addressing bioactive compound metabolism are needed to improve the production of secondary metabolites for pharmaceutical applications.

## Functional gene mining of medicinal plant transcriptome to characterize secondary metabolite biosynthesis pathways

Phenolic compounds, terpenoids and alkaloids are important secondary metabolites in medicinal plants (Mrudulakumari Vasudevan and Lee, 2020), but the pharmacological activity of medicinal plants is often estimated by the content of phenolic compounds. Phenolic compounds consist of (1) monophenols (*e.g.*, benzoic acid derivatives (hydroxybenzoic acids)) and cinnamic acid derivatives (*e.g.*, hydroxycinnamic acids); (2) oligophenols (*e.g.*, flavonoids, stilbenes and coumarins) and (3) polyphenols (*e.g.*, lignin and tannins) (Marchiosi et al., 2020). Due to the broad benefits of phenolic compounds to human health, current research aim at improving the production of phenolic compounds on a large scale.

**Figure 2:**
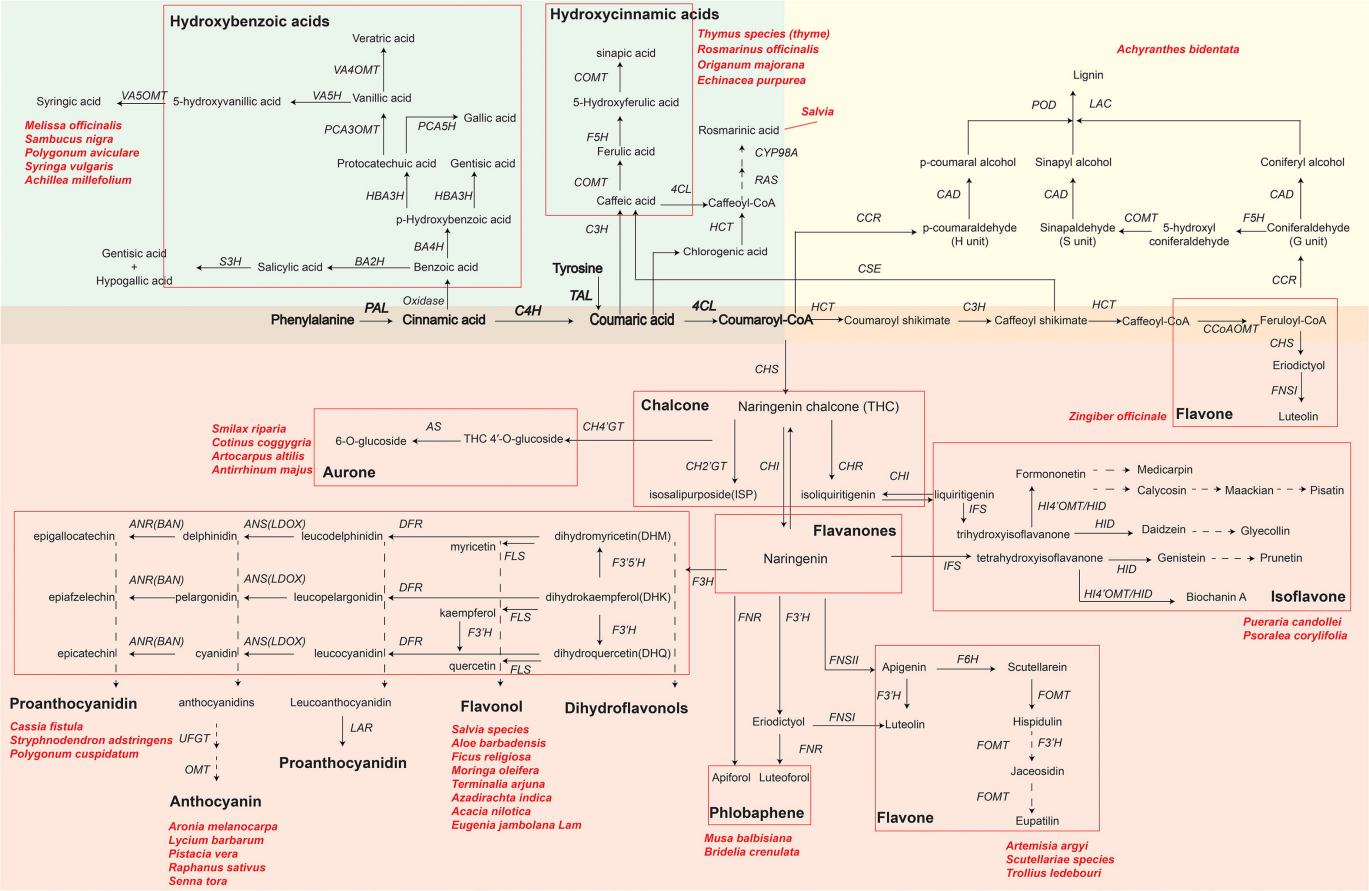
Key genes and metabolites of three phenolics compounds biosynthesis pathways in plants. The green, yellow and red background areas represent the biosynthetic pathways of phenolic acids, lignin and flavonoids, respectively. Bolded metabolites and genes represent the beginning of henylpropanoid pathway shared by phenolics compounds biosynthesis. Different flavonoids are indicated by black bold font and grouped by red rectangular boxes in each pathway. Key genes in biosynthesis pathways are in italics: *PAL*, phenylalanine ammonialyase; *C4H*, cinnamic acid 4-hydroxylase; *4CL*, 4-coumarate-CoA ligase; *HCT*, hydroxycinnamoyl CoA shikimate hydroxycinnamoyl transferase; *RAS*, rosmarinic acid synthase; *CYP98A*, cytochrome P450-dependent monooxygenase; *C3H*, *p*-coumarate 3-hydroxylase; *CHS*, chalcone synthase; *CH4’GT*, chalcone 4’-O-glucosyltransferase; *CH2’GT*, chalcone 2′-glucosyltransferase; *AS*, aureusidin synthase; *CHR*, chalcone reductase; *CHI*, chalcone flavanone isomerase; *IFS*, isoflavone synthase; *F3′H*, favonoid 3′ hydroxylase; *FNS* (*FNSI* and *FNSII*), flavone synthase; *F6H*, flavanone-6-hydroxylase; *FOMT*, flavonoid O-methyltransferase; *HI4’OMT*, hydroxyisoflavone 4’-O-methyltransferase; *HID*, hydroxyisoflavanone dehydratase; *DFR*, dihydroflavonol 4-reductase; *F3H*, favanone 3-hydroxylase; *F3’5’H*, flavonoid-3′,5′-hydroxylase; *FLS*, flavonol synthase; *ANS*, anthocyanidin synthase (*LDOX*, leucoanthocyanidin dioxygenase); *ANR*, anthocyanidin reductase; *LAR*, leucoanthocyanidin reductase; *UFGT*, UDP glucose:flavonoid 3-O-glycosyltranferase; *OMT*, O-methyltransferase; *CCoAOMT*, caffeoyl-CoA O-methyltransferase; *F5H*, ferulate 5-hydroxylase; *CSE*, caffeoyl shikimate esterase; *COMT*, caffeic acid O-methyltransferase; *CCR*, cinnamoyl-CoA reductase; *CAD*, cinnamyl alcohol dehydrogenase; *LAC*, laccase; *POD*, peroxidase; *TAL*, tyrosine ammonia lyase; *BA2H*, benzoic acid 2-hydroxylase; *S3H*, salicylic acid 3-hydroxylase; *BA4H*, benzoic acid 4-hydroxylase; *HBA3H*, p-hydroxybenzoic acid 3-hydroxylase; *PCA5H*, protocatechuic acid 5-hydroxylase; *PCA3OMT*, protocatechuic acid 3-O-methyltransferase; *VA4OMT*, vanillic acid 4-O-methyltransferase; *VA5H*, vanillic acid 5-hydroxylase; *VA5OMT*, vanillic acid 5-O-methyltransferase. Naringenin chalcone and naringenin in a larger font are key primarily intermediate metabolite in flavonoid biosynthesis. Dashed arrows indicate that some unknown enzymes are involved in these processes. Scientific names are representative medicinal plants, which are exploited by various pharmaceutical companies to produce phenolics compounds.

Using RNA-seq technology can identify key functional genes or transcription factors involved in phenolic compound biosynthetic pathways of medicinal plants. With an improved understanding of phenolic compound biosynthesis pathways, pharmaceutical industries can achieve the goal of obtaining bioactive compounds more efficiently. Here, we summarize the synthetic metabolic pathways of three representative phenolic compounds: flavonoids, phenolic acids, and lignin (**Figure 2**) by showing key genes and metabolites as well as medicinal plants that have been reported to synthesize specific metabolites. As shown in **Figure 2**, biosynthesizing phenolic compounds starts from the phenylpropanoid pathway, where phenylalanine (or tyrosine) is subsequently converted to the intermediates cinnamic acid, coumaric acid and p-coumaroyl-CoA by phenylalanine ammonia lyase (PAL; or tyrosine ammonia lyase, TAL), cinnamate 4-hydroxylase (C4H), and 4-coumaroyl coenzyme A (CoA) ligase (4CL). These intermediates serve as a starting compound(s) to further produce other metabolites in each synthetic metabolic pathway.

### Phenolic acid biosynthetic pathway

Phenolic acids, as representative compounds of monophenols, have gained a lot of attentions because of their antioxidant, antimicrobial, anti-inflammatory activities. In commercial Danshen (*Salvia*) decoctions, phenolic acids, especially salvianolic acid, are the major marker component used for quality assessment according to the official Chinese Pharmacopoeia. A proposed biosynthetic pathway of phenolic acids in *Salvia apiana* has been schemed based on full-length transcriptomic and metabolomic profiling (Shi et al., 2021; Hu et al., 2022). In addition, functional genes and transcription factors as regulators of phenolic acids biosynthesis in *Salvia miltiorrhiza* have been reported (Yu et al., 2018; Deng et al., 2020; Zhou et al., 2021c). For example, rosmarinic acid synthase (RAS) and a cytochrome P450-dependent monooxygenase (CYP98A), precursors responsible for rosmarinic acid biosynthesis, were mostly highly expressed in roots. MYB transcription factors have a positive effect on methyl jasmonate (MeJA)-induced phenolic acid biosynthesis in *S. miltiorrhiza*. Overexpressing *SmMYB2* in hairy roots significantly increased the levels of salvianolic acids by binging the MYB-binding motifs of CYP98A14 and upregulating CYP98A14 expression.

### Lignin biosynthetic pathway

Medicinal plant lignin are reported as antioxidants (Karmanov et al., 2021; Lu et al., 2022). Lignin and their degradation products (*e.g.*, phenylpropanoids) have shown prominent anti-UVC (ultraviolet C) activities (Sakagami et al., 2022). In the terminal of lignin biosynthesis, peroxidase (POD) catalyzed individual monolignols to polymerize the complex lignin (**Figure 2**). The comparison between the CM (consecutive monoculture) and NG (normal growth for 1 year) root transcriptomes of *Achyranthes bidentata* revealed that genes encoding POD were mostly activated in the CM condition, suggesting the contribution of POD to lignin biosynthesis pathway (Yang et al., 2018). Comparing to the number of research in the area of phenolic acids in medicinal plants, research that focus on exploring lignin synthetic pathway from medicinal plants are limited.

### Flavonoids biosynthetic pathway

Flavonoids, such as flavone, flavonol, flavanones, anthocyanin, chalcone, aurone, isoflavone and proanthocyanidin, are the most widely distributed phenolic compounds in plants (Liu et al., 2021c). Starting from coumaroyl-CoA, biosynthesis pathways of flavonoids are further divided into different branches responsible for the accumulation of various flavonoids, under the regulation of different enzymes and genes (**Figure 2**). For example, the expression of chalcone synthase (CHS), the key and first rate-limiting enzyme in the flavonoid biosynthetic pathway, decides the total content of flavonoids in medicinal plants (Yang et al., 2019; Ohta et al., 2021). In addition, two types of flavone synthase (FNS), FNSI (soluble 2-oxoglutarate-dependent dioxygenases) and FNSII (NADPH-dependent cytochrome P450 monoxygenases), play important roles in the accumulation of luteolin and apigenin in different medicinal plants (Zhao et al., 2016; Jiang et al., 2019; Li et al., 2020a; Tian et al., 2022).

Plant-derived alkaloids have been used as medicine for a long time. Alkaloids are a large and complex group of cyclic compounds that contain nitrogen. Medicinal plants from Ranunculales, such as opium poppy (*Papaver somniferum*) and *Rhizoma Coptidis*, are often used as model systems for studying benzylisoquinoline alkaloids (BIAs) biosynthesis. Gene mining by functional transcriptomics can effectively promote the discovery of alkaloids biosynthetic pathway and facilitate their characterization (Li et al., 2020c; Hao et al., 2021; Xu et al., 2022). Multicopy genes in alkaloids pathway, such as BBE and 4OMT, were divergently expressed between copies in *P. somniferum*, being differentially expressed between either tissues or different stages, suggesting possible differences in their regulatory function (Pei et al., 2021). Wang et al. reported that the total alkaloids contents in Protocorm-like bodies (PLBs) was almost twice as high as that of plant organs of *Dendrobium officinale*, a species with high alkaloid content. Using RNA-seq technology, the authors identified putative genes that encode enzymes in the alkaloids biosynthetic pathway in PLBs and leaves of *D. officinale* (Wang et al., 2021).

The effects of plant growth duration on bioactive substance accumulation in the same medicinal plant species were explored (Lin et al., 2020; Liu et al., 2022). For example, comparing transcriptome profiles of one to four years old *D. officinale* stems revealed that key genes, such as *CHS* and *FLS* that are involved in flavonol synthesis, were highly expressed in the biennial samples, suggesting that the optimal harvesting period of *D. officinale* is 2-3 years (Yuan et al., 2022). Moreover, multiple DEGs involved in liquiritin biosynthesis (e.g., UDP-glucosyltransferase (UGTs)), displayed distinct expression patterns in *Glycyrrhiza uralensis* farmed between 1 year and 3 years (Zhong et al., 2022).

It is of a great significance to characterize genes related in secondary metabolite biosynthesis between closely related species or varieties in medicinal plants (Koo et al., 2022; Liu et al., 2022; Shafi et al., 2022), or between different tissues in same species (He et al., 2022a; Naika et al., 2022). The comparative transcriptome analysis of the marijuana strain Purple Kush and the ‘Finola’ cultivar of *Cannabis sativa* demonstrated that the Δ9-tetrahydrocannabinolic acid synthase was detected in Purple Kush but replaced by cannabidiolic acid synthase in the ‘Finola’ cultivar, explaining the significant chemical difference between marijuana-derived Δ9-tetrahydrocannabinol (THC) and hemp-derived cannabidiol (CBD) (van Bakel et al., 2011). Moreover, transcriptional changes during tanshinones accumulation stage among different varieties of *Salvia miltiorrhiza* expanded our vision on intraspecific variation and gene regulation mechanism of secondary metabolite biosynthesis pathways (Zhou et al., 2021b). Interestingly, platycodin D content of calli was higher than that of leaves in *Platycodon grandiflorus*, while platycoside E content of calli was lower than that of leaves. Comparative analysis of the transcriptome data identified 54 and 23 specifically expressed transcription factors in leaf and calli respectively, providing valuable resources for researchers to study the conversion between platycoside E and platycodin D (Su et al., 2021). Li et al. used RNA-seq to study the molecular mechanisms of different tissues of Chinese sage (*Salvia miltiorrhiza*) in response to moderate drought stress. GO enrichment analysis showed that several transcription factors, such as AP2/ERF, bHLH and WRKY that regulate abiotic responses of *S. miltiorrhiza*, were significantly enriched in roots and leaves. Under moderate drought stress, genes encoding key enzymes in the biosynthesis of phenylpropane and terpenoids were also upregulated (Li et al., 2020d). Together, these results provide us a solid foundation to further investigate biosynthesis mechanisms of medicinal components in medicinal plants.

## Proteomic dissection of medicinal plants in different manners for potential drug development

**Figure 3:**
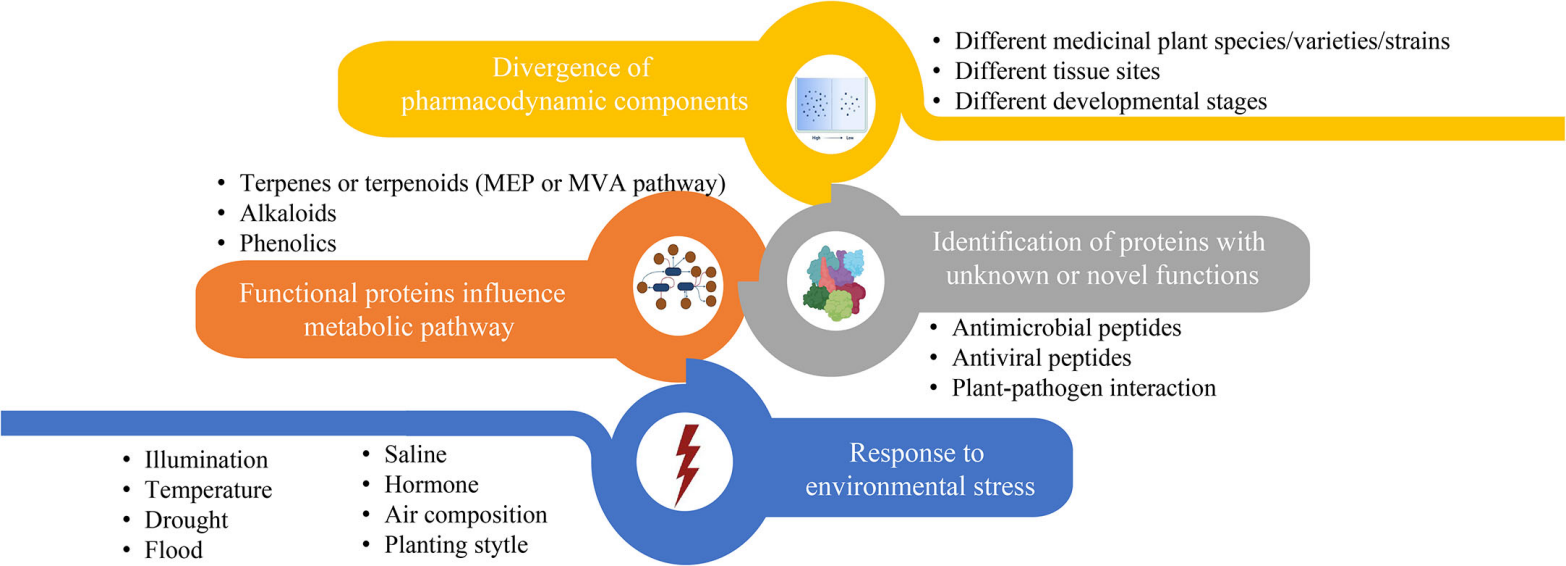
The main contents of proteome research related to biosynthesis or accumulation of metabolites in medicinal plant.

Over the past decades, considerable amounts of medicinal plants have been proved to exhibit potent effects on different human diseases based on their specific therapeutic compounds. **Figure 3** summarizes the main research foci of medicinal plant proteomics that lead to the identification of proteinaceous compounds involved in active bioactive compound conversion in recent years. The contents of these four foci often complement each other and are not studied independently. For example, global proteome and phosphoproteome profilings of *Dendrobium huoshanense* under greenhouse planting (GP) and the cultivation modes of stone planting under the forest (SPUF) revealed that SPUF was more conducive to the accumulation of polysaccharides and alkaloids, and that there was a possible correlation between phosphorylation levels of different enzyme sites and the polysaccharide/alkaloid content (Wu et al., 2022b).

Most of proteomic studies in medicinal plants focus on investigating protein abundance changes under different environmental conditions (**Figure 3**, blue part). For instance, illumination (Zhang et al., 2021), temperature, drought (Xu et al., 2021; Zhang et al., 2022a), flood, saline (Fortini et al., 2022), exogenous hormone (Yang et al., 2022), air composition and planting style are the main factors affecting the content of enzymes or regulators involved in metabolites biosynthesis in the medicinal plants. Unlike abiotic conditions, studies that investigate the effects of biotic stresses on the proteins influencing accumulation of active compounds in traditional medicinal plants are relatively less. Through leaf and rhizome proteomic analysis of kutki (*Picrorhiza kurroa*), the abundance of proteins associated with carbon metabolism in CO_2_ enhancement were upregulated (*i.e.*, glucose and fructose) in a tissue-specific manner (Kumar et al., 2020). Two-dimensional electrophoretic and MALDI-TOF/TOF mass spectrometry analyses identified 20 differentially expressed proteins of hoary cress (*Lepidium draba*) related to photosynthesis, energy metabolism and other functions to water stress (6% PEG) (Jamshidi Goharrizi et al., 2020). Many precious medicinal plants grow in high latitudes are cold tolerant. Exploring heat responses at the protein level between heat-tolerant and heat-sensitive strains of *Clematis florida* provided evidence on its adaptive mechanism of thermotolerance (Jiang et al., 2020). *Sophora alopecuroides* is a famous saline-alkali tolerant and drought-tolerant medicinal plant. Tandem mass tag (TMT) based proteomic profiling of *S. alopecuroides* leaves confirmed that salt stress altered several transporter proteins related to the secondary metabolite’s biosynthesis pathway in *S. alopecuroides* leaves (Ma et al., 2022).

Other proteomic studies identified novel proteins/peptides of medicinal plant origin with pharmacological interests (**Figure 3**, grey part) (Moyer et al., 2021b). For instance, seven novel peptides belonging to three antimicrobial peptide classes, including lipid transfer proteins, snakins and a defensin, were identified by MS-based peptidomics analysis from the aerial tissues of edible amaranth (*Amaranthus tricolor*) plants (Moyer et al., 2021a). Two novel bioactive peptides isolated from the Asian medicinal plant *Acacia catechu* are recommended for further investigation as antiviral peptides with their potent inhibition activities against dengue viruses (Panya et al., 2019).

**Figure 4:**
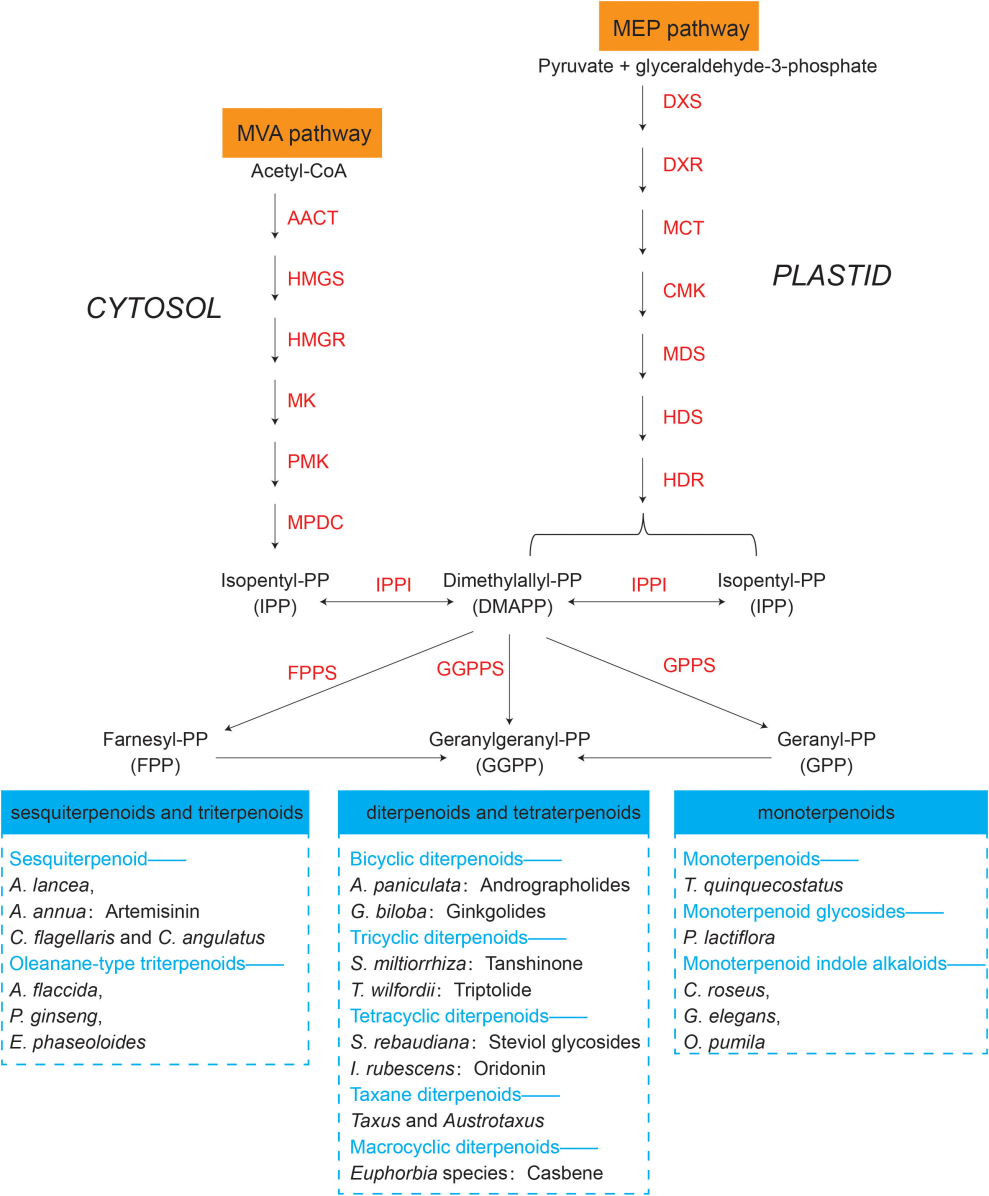
Terpenoids synthesized by representative medicinal plants through mevalonate (MVA) and methylerythritol phosphate (MEP) pathways. The key proteins involved in the two pathways are in orange. AACT, acetoacetyl-CoA thiolase; HMGS, 3-hydroxy-3-methylglutaryl-CoA synthase; HMGR, 3-hydroxy-3-methylglutaryl-CoA reductase; MK, mevalonate kinase; PMK, phosphomevalonate kinase; MPDC, mevalonate diphosphate decarboxylase; DXS, 1-deoxy-d-xylulose 5-phosphate synthase; DXR, 1-deoxy-d-xylulose 5-phosphate reductoisomerase; MCT, 2C-methyl-d-erythritol 4-phosphate cytidyl transferase; CMK, 4-diphosphocytidyl-2C-methyl-d-erythritol kinase; MDS, 2C-methyl-d-erythritol 2,4-cyclodiphosphate synthase; HDS, 1-hydroxy-2-methyl-2-€-butenyl 4-diphosphate synthase; HDR, 1-hydroxy-2-methyl-2-(E)-butenyl 4-diphosphate reductase; IPPI, Isopentenyldiphosphate-isomerase; FPPS, farnesyl diphosphate (FPP) synthase; GGPPS, geranylgeranyl diphosphate (GGPP) synthase; GPPS, geranyl diphosphate (GPP) synthase. Representative medicinal plants containing various terpenoids are listed in the blue dashed box.

The synthesis regulatory pathway of secondary metabolites involves many functional enzymes which provide guidance for drug discovery based on protein expression (**Figure 3**, orange part). In addition to phenolic compounds and alkaloids in medicinal plants and their corresponding biosynthesis pathways that we previously discussed (**Figure 2**), terpenoids are of economic interests for drug development. The terpenoid metabolites are derived from the common precursor isopentenyl diphosphate (IPP), which can be synthesized *via* two different pathways: the mevalonate (MVA) pathway in the cytoplasm and the methylerythritol phosphate (MEP) pathway in plastids. Terpenoids can be classified into monoterpenes, sesquiterpenes, meroterpenes, triterpenes, diterpenoids and other terpenoids in medicinal plants (Awouafack et al., 2013; Sandjo and Kuete, 2013b; Sandjo and Kuete, 2013a; Tchimene et al., 2013; Nazir et al., 2021) (**Figure 4**). Among the differentially expressed proteins between leaves and rhizomes of soft windflower (*Anemone flaccida*), most proteins involved in the metabolic pathway of triterpenoid saponins biosynthesis were upregulated in rhizomes (Zhan et al., 2016). Comparative proteome analysis of the leaves, roots, shoots and fruits of Korean Ginseng by label-free quantitative proteomics identified that 67 out of 1,179 differentially regulated proteins were associated with ginsenoside biosynthesis pathways, including MEP pathway, MVA pathway, UDP-glycosyltransferase and oxidoreductase (Van Nguyen et al., 2021).

Divergence of pharmacodynamic components in different species/varieties/strains (Qin et al., 2014; Song et al., 2021), tissues (Guo et al., 2022; Pan et al., 2022) and development stages (Huang et al., 2021; Zhang et al., 2022b) of medicinal plants implicates expression level of key proteins involved in biosynthesis and metabolism of medicinal compounds (**Figure 3**, yellow part). Based on quantitatively targeted subproteomic analysis of the high and low artemisinin content of sweet wormwood (*Artemisia annua*), the increase expression of DBR2 accounts for the high artemisinin content (Chen et al., 2020). The genus *Paris* includes a variety of genotypes with different medicinal contents. Proteomic changes in rhizomes between closely related species of *Paris polyphylla* enhanced our understandings on the molecular basis of different medicinal properties. Such as, the higher efficiency of sucrose utilization in the sugar metabolic pathway of *P. polyphylla* var. *chinensis* fare probably related to the elevated protein abundance (Liu et al., 2019). Comparing protein contents of latex at different developmental stages revealed that stress- and defense-related proteins in green fruit phase were of higher abundances, but upregulated proteins in flowering phase were related to transcription, protein folding, and active transport of molecules, providing new insights into the biology and medicinal use of greater celandine (*Chelidonium majus*) (Nawrot et al., 2017).

## Metabolomics profiles of medicinal plants revealed that both primary and secondary metabolites have pharmacological potential

**Figure 5:**
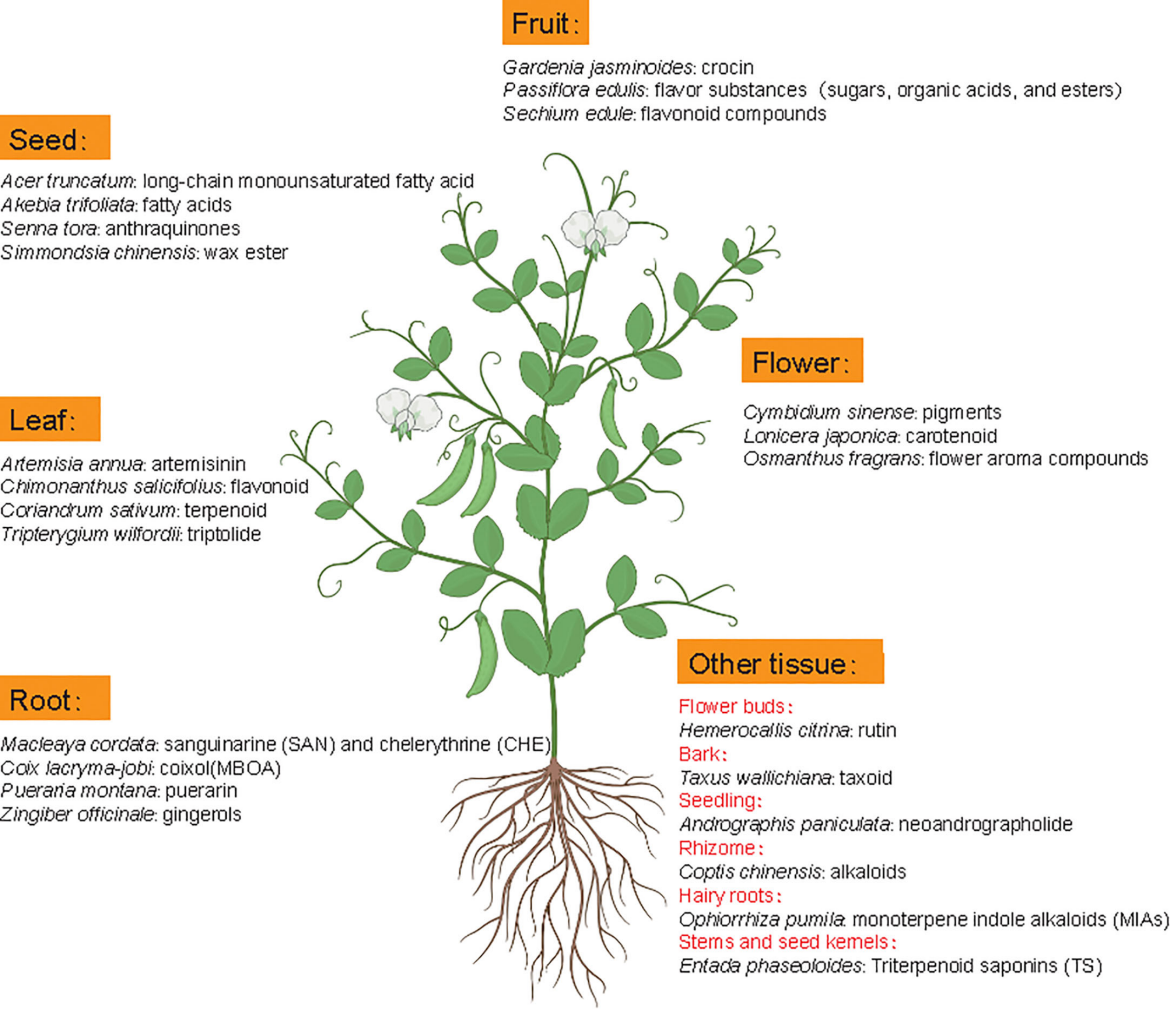
Metabolic components with main pharmacodynamic function from different tissues of representative medicinal plants. Latin names are the representative medicinal plants with specific tissue(s) as the main source of medicinal ingredients.

Metabolomics studies in medicinal plants aim to provide comprehensive examination of metabolite profiles and quality assessment of medicinal plants. Although the extraction method could influence the effective acquisition of metabolites, the difference of medicinal material quality is mainly attributed to metabolite diversity and composition among different tissues or species because roots, leaves, flowers, fruits, seeds, rhizomes, bark or whole plants of many natural plants can contain different active ingredients with nutritional or therapeutic function (**Figure 5**) (Rai et al., 2021). For example, artemisinin mainly accumulates in leaves of *Artemisia annua* but gingerols is mainly stored in *Zingiber officinale* roots. In some cases, active compounds accumulate in special tissues, like bark (*Axus wallichiana*: taxoid) and rhizome (*Coptis chinensis*: alkaloids).

To comprehensively characterize metabolites and low-molecular-weight molecules with therapeutic values, we often adapt two technology platforms: nuclear magnetic resonance (NMR) and/or gas/liquid chromatography-mass spectrometry (GC-MS or LC-MS/MS). For example, GC-MS and LC-MS/MS generated metabolic profiles of roots, stems, and leaves of *Panax notoginseng* from different geographical regions showed that the composition of saponins was similar between root and stem but was different than plant leaves. These results provide further evidence that different parts in addition to *P. notoginseng* roots, for example their stems, can be used in practice (Gao et al., 2022). Another investigation on metabolite profiles of twenty medicinal plants identified that two hydroxylated fatty acids (13S-Hydroxy-9Z,112,15Z-octadecatrienoic acid and 13-Hydroxy-9Z,11E-octadecadienoic acid) are potential targets for developing anti-viral drugs (More et al., 2022). Similarly, metabolome analysis of neuroactive plants (*e.g.*, *Hypericum perforatum* L. (St. John’s wort), *Passiflora incarnate* L. (maypop), *Valeriana officinalis* L. (Valerian) and *Melissa officinalis* L. (Lemon balm)) revealed that primary metabolites in the tricarboxylic acid (TCA) cycle as well as secondary metabolites belong to flavonoids and terpenoids positively correlated with BDNF (*i.e.*, brain-derived neurotrophic factor; an important indicator of neurodegenerative diseases) expression level *in vitro* (Gonulalan et al., 2020).

Technological advances in mass spectrometry-based platforms have facilitated the separation and identification of multiple metabolomic profiles of medicinal plants. With the implementation of the Herbal Genome Project (Chen et al., 2011; Su et al., 2022) and the development of traditional Chinese medicine synthetic biology (Mortimer, 2019), the metabolomic research of medicinal plants will accelerate the discovery of novel bioactive compounds with pharmacological potential (Bhardwaj et al., 2022; Nisar et al., 2022).

## Prospects

**Figure 6:**
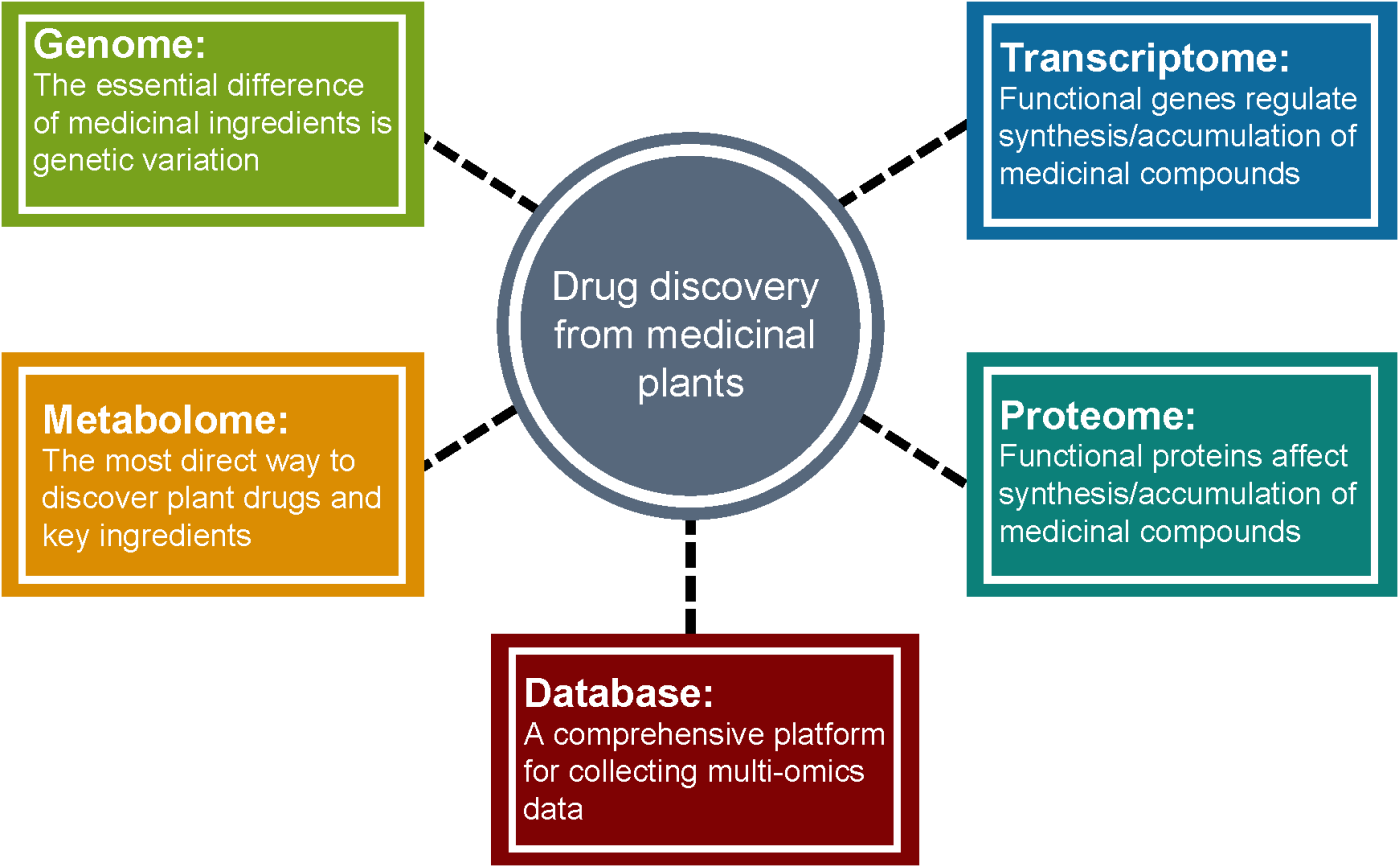
Medicinal plant multi-omics research to facilitate drug discovery.

Medicinal plants have significant economic and social benefits due to their pharmacological activities. The increase of habitat destruction and human consumption of medicinal plants worldwide has increased their risk of extinction (Wang et al., 2020). However, unlike agricultural/horticultural crops, traditional breeding of medicinal plants is a more challenging task because factors such as medicinal parts, production of active ingredients with pharmacological potential, and growth cycle need to be considered. Multi-omics analysis coupled with bioinformatics and statistical analysis is a comprehensive approach to uncover the chemical diversity and the regulatory mechanisms and the formation of pharmacological properties of medicinal plants (**Figure 6**). Once genes, metabolites, peptides, or proteins involved in the biosynthetic pathways of active medicinal plant bio-compounds are elucidated, genome engineering or synthetic biology can be applied to produce them effectively and sustainably. Therefore, incorporating multi-omics technologies into medicinal plant research should be encouraged by organizations and research institutions across the globe to promote their cultivation in order to fulfill the needs of synthesizing bioactive components of medicinal plants for pharmaceutical applications.

## Author contributions

WZ conceived the manuscript, and CL and JW supervised the study. WZ, YZ, MJ, and YL wrote the paper. WZ, MJ, and YL analyzed data, and generated figures and tables. All authors reviewed the manuscript and approved the submitted version.
